# Nitric oxide signalling and neuronal nitric oxide synthase in the heart under stress

**DOI:** 10.12688/f1000research.10128.1

**Published:** 2017-05-23

**Authors:** Yin Hua Zhang

**Affiliations:** 1 Department of Physiology & Biomedical Sciences, College of Medicine, Seoul National University, 103 Dae Hak Ro, Chong No Gu, 110-799 Seoul, Korea, South; 2 Yanbian University Hospital, Yanji, Jilin Province, 133000, China; 3 Division of Cardiovascular Sciences, School of Medical Sciences, Faculty of Biology, Medicine and Health, University of Manchester, Manchester, UK

**Keywords:** nitric oxide, neuronal nitric oxide, cardiac protection, heart disease

## Abstract

Nitric oxide (NO) is an imperative regulator of the cardiovascular system and is a critical mechanism in preventing the pathogenesis and progression of the diseased heart. The scenario of bioavailable NO in the myocardium is complex: 1) NO is derived from both endogenous NO synthases (endothelial, neuronal, and/or inducible NOSs [eNOS, nNOS, and/or iNOS]) and exogenous sources (entero-salivary NO pathway) and the amount of NO from exogenous sources varies significantly; 2) NOSs are located at discrete compartments of cardiac myocytes and are regulated by distinctive mechanisms under stress; 3) NO regulates diverse target proteins through different modes of post-transcriptional modification (soluble guanylate cyclase [sGC]/cyclic guanosine monophosphate [cGMP]/protein kinase G [PKG]-dependent phosphorylation, *S*-nitrosylation, and transnitrosylation); 4) the downstream effectors of NO are multidimensional and vary from ion channels in the plasma membrane to signalling proteins and enzymes in the mitochondria, cytosol, nucleus, and myofilament; 5) NOS produces several radicals in addition to NO (e.g. superoxide, hydrogen peroxide, peroxynitrite, and different NO-related derivatives) and triggers redox-dependent responses. However, nNOS inhibits cardiac oxidases to reduce the sources of oxidative stress in diseased hearts. Recent consensus indicates the importance of nNOS protein in cardiac protection under pathological stress. In addition, a dietary regime with high nitrate intake from fruit and vegetables together with unsaturated fatty acids is strongly associated with reduced cardiovascular events. Collectively, NO-dependent mechanisms in healthy and diseased hearts are better understood and shed light on the therapeutic prospects for NO and NOSs in clinical applications for fatal human heart diseases.

## Introduction

**Figure 1.**
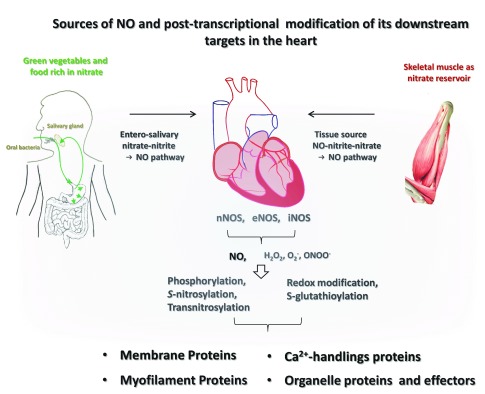
Schematic diagram demonstrating the sources of nitric oxide (NO) in the heart and mechanisms mediating the effects of NO and its derivatives. Both exogenous sources (nitrate-rich vegetables and food through the entero-salivary nitrate–nitrite–NO pathway and skeletal muscle nitrate → NO pathway) and endogenous sources (neuronal nitric oxide synthase [nNOS], endothelial NOS [eNOS], or inducible NOS [iNOS]) determine the bioavailable NO in the myocardium. NO regulates downstream targets through soluble guanylate cyclase (sGC)/cyclic guanosine monophosphate (cGMP)/protein kinase G (PKG)-dependent phosphorylation, S-nitrosylation, and transnitrosylation. Alternatively, NOS-derived radicals and NO-related derivatives (H _2_O _2_, O _2_^–^, and peroxynitrite [ONOO ^–^], etc.) affect downstream effectors through the oxidation and *S*-glutathionylation. As such, NO regulates membrane proteins, Ca ^2+^-handling proteins, membrane proteins, and organelle effectors in the cardiac myocytes.

Nitric oxide (NO) is an essential molecule that plays fundamental roles in maintaining cardiovascular functions in animals and humans ^1– 5^. The NO that exerts biological functions in the myocardium can be acquired through exogenous sources or is produced from the endogenous endothelial and neuronal NO synthases (eNOS and nNOS, respectively, which are constitutively expressed in the myocytes) and from inducible NOS by inflammatory cytokines following infection ^4– 6^ ( Figure 1). In the last few decades, our understanding of the detailed mechanisms, the effects of NO on myocardial functions, and the roles for NOSs in diseased hearts has improved. Comprehensive approaches have been undertaken to achieve this outcome, including manipulation of the upstream and downstream effectors of NOSs (pharmacologically or genetically modified NOS regulation and viral infections of specific NOS genes), supplementation of NO mimetics (for example, exogenous NO donors or NO substrates), and systematic detection of plasma and tissue NO ^4, 5, 7, 8^. Nevertheless, the practical implications of NO and its precursors or regulators in translational and therapeutic strategies in cardiovascular diseases are hampered because of the complex nature of NO and the array of downstream signalling cascades and effectors in the myocardium. In the initial part of this review, I will provide a systematic overview of the sources and post-transcriptional modification of NO in the myocardium; the latter part of the review will focus on recent advances of nNOS-targeting proteins and responses in the heart under stress. Ultimately, I will delineate the prospect for using the therapeutic platform of nNOS and NO to target human heart diseases.

## Exogenous sources of nitric oxide

It is generally acknowledged that NO is derived from the classic ** L-arginine–NOS–NO pathway. In fact, NO that exerts functions in the myocardium may also be acquired from the alternative source of NO, the nitrate–nitrite–NO pathway ** ( Figure 1). Nitrate (NO _3_^−^) in various types of green leafy vegetables and food ^9– 11^ is taken up into the plasma to become a reliable reservoir and the stable precursor of NO (the half-life of nitrate in the plasma is 5–6 hours). Nitrate from this source is actively taken up by the salivary gland, is secreted in concentrated form in the saliva (about 10-fold that in the plasma), and is subsequently reduced to more active nitrite (NO _2_^−^) in the oral cavity by nitrate reductases of commensal bacteria (entero-salivary NO pathway). Nitrite is reduced to NO in the acidic environment of the stomach and is absorbed into the blood in the upper gastrointestinal system (the half-life of nitrate in the plasma is about 30 minutes), while the rest mixes with the nitrite formed from nitrate derived from endogenous NOS-produced NO. Various enzymes and proteins are known to be involved in the NO metabolite cycle and nitrite’s reduction to NO, including xanthine oxidase ^12, 13^, deoxyhaemoglobin and deoxymyoglobin ^14– 16^, neuroglobin ^17^, respiratory chain enzymes ^18^, cytochrome P450 ^19^, aldehyde oxidase ^20^, carbonic anhydrase ^21^, and NO synthase ^22^. Overall, about 25% of nitrate undergoes re-uptake by the salivary gland and produces functional NO in the circulation; the rest of the nitrate is eventually excreted in the urine. The amount of NO from exogenous sources can be as high as the amount that is produced from NOSs in the tissues (with enough daily consumption of green leafy vegetables or food, nitrite intake varies from 0 to 20 mg/day ^23^), indicating the importance of this pathway in supplementing local NO in the tissue. Notably, unlike NO from the L-arginine–NOS–NO pathway, food-derived functional NO is oxygen independent ^10, 11^. Accordingly, NO from this source becomes more important in ischaemic or hypoxic conditions, such as myocardial infarction, hypertrophy, and heart failure.

NO or nitrite can undergo an oxidative process via oxyhaemoglobin or oxymyoglobin to produce stable nitrate, which can be reduced back to nitrite and NO by molybdopterin-containing mammalian nitrate reductases, such as xanthine oxidoreductase or aldehyde oxidase ^10, 11^. Therefore, there is a constant recycling of NO precursors, metabolites, and NO that maintains exogenous NO in the human body. The respective contributions of the endogenous versus exogenous NO to intracellular signalling and function in healthy and diseased hearts *in vivo* remain to be revealed.

A number of organs or tissues (for example, neurons, liver, heart, skeletal muscle, kidney, arteries such as the aorta, and endothelium) are the active sites for NO production from constitutive NOSs. Recently, it has been shown that skeletal muscle is a dynamic nitrate reservoir that increments plasma nitrate and nitrite because of the abundance of the tissue in the mammalian body ^24^. nNOS in the skeletal muscle contributes to the supply because it is the only isoform in the skeletal muscle ^25^. However, the proportions of NO from the specific sources that contribute to the bioavailable NO in the myocardium remain undetermined.

## Endogenous sources of nitric oxide in the myocardium

### Endothelial nitric oxide synthase

Classically, eNOS is the primary isoform of NOS that plays important roles in NO regulation of physiological functions in the majority of tissues, including the heart ^4, 5, 7, 8^. In the cardiac myocyte, eNOS is located in spatial microdomains of the plasma membrane (caveolae and lipid rafts), Golgi apparatus, nucleus, and mitochondria ^8, 26^. eNOS displays the highest activity at the plasma membrane, followed by outer membranes of the cis-Golgi and low activity in the cytosol, nucleus, and mitochondria ^26, 27^; therefore, localisation is the main determinant of eNOS activity for specific biological functions. Conversely, mis-localisation of eNOS has been shown to reduce its capacity to generate NO in intact cells ^26– 28^. Post-translational cysteine palmitoylation (Cys ^15^ and Cys ^26^) or N-myristoylation at Gly ^2^ of eNOS, catalysed through Asp-His-His-Cys motif-containing palmitoyl acyltransferases, is critical in locating eNOS to the membrane for optimising its activity ^28, 29^.

A number of alternatively spliced eNOS variants have been identified: specifically, eNOS lacking exons 20 and 21 ^30^ or three splice variants of eNOS containing novel 3' splice sites within intron 13 ^31^. These alternative splicing variants produce truncated isoforms of eNOS with maintained ^30^ or diminished ^31^ NO-producing activity. Reduced eNOS activity has been shown in the heterodimer of eNOS (the splice variants with the full-length eNOS) ^31^, although the existence of the splice variants of eNOS and their functional impact in the myocardium are unclear. In diseased hearts, the protein expression and the activity of eNOS are known to be downregulated ^32– 34^ or uncoupled ^35^. These results suggest that maintaining eNOS protein and activity in its “coupled form” is beneficial by preventing the early stage of disease progression in the heart.

### Neuronal nitric oxide synthase

Recent consensus is that nNOS is the isoform that plays the principal role in cardiac physiology and pathology because nNOS is expressed in all parts of the heart, including the autonomic nervous system innervating the heart, aortic and pulmonary arteries, coronary artery, and the atrial and ventricular myocardium ^7, 8^. As such, nNOS is well placed to fill essential roles in modifying sympathetic and parasympathetic tones, controlling heart rate, delivering essential nutrients through coronary arteries, and regulating myocardial contractility. In the myocardium, nNOS is predominantly localised in the sarcoplasmic reticulum (SR) ^6^ and is involved in the Ca ^2+^ handling processes of cardiac excitation-contraction coupling ^7, 8^. In addition, nNOS interacts with α-syntrophin through the scaffolding protein postsynaptic density-95 (PSD95) via the PSD-95/Discs large/ZO-1 homology domain (PDZ domain) and forms a multi-protein complex with the plasma membrane Ca ^2+^ pump (PMCA) and voltage-gated Na ^+^ channel (Nav1.5) ^36^. In addition, nNOS binds to its PDZ-binding motif to direct nNOS to the subcellular compartments, as is the case of nNOS in the nucleus ^37^, which regulates the transcription and activation of the elements required for oxidative phosphorylation and mitochondrial biogenesis ^37^. Recently, we have shown that nNOS is upregulated in the myocardium from the early stage of disease progression (e.g. hypertension ^34^) and facilitates lusitropy through myofilament Ca ^2+^ desensitisation ^34^.

Until recently, most of the responses of nNOS were attributed to nNOSα or nNOSμ ^7, 8^. However, the existence of various splice variants of nNOS (nNOSβ, nNOSγ, and nNOS2) suggests that splice variants of nNOS may be involved in producing NO and regulating contractile function in the heart. Very recently, we have presented novel evidence to show that nNOSβ, which does not possess the PDZ domain, is expressed in the myofilament fraction of cardiac myocytes from the hearts of healthy and hypertensive rats ^38^. These results indicate that nNOSβ may play important roles in cardiac myofilament. On the other hand, it has been documented in skeletal muscle that nNOSβ is functionally expressed in the Golgi apparatus and mediates myofilament regulation during exercise ^25^. A comprehensive understanding of nNOS and its splice variants in the organelles and their roles in cardiac function and protection in the healthy and diseased hearts remains to be explored.

Notably, the co-existence of eNOS and, nNOS and their splice variants in the myocardium and their translocation, transcription, and post-translational modifications underlie the complex scenario of NO in the heart ^7, 8, 39^, more so under pathological stress. This is represented by a contrasting tendency in protein expression and activities of eNOS and nNOS in the failing myocardium or in the hypertensive heart; that is, eNOS protein expression is reduced significantly, whereas nNOS protein expression and activity are increased ^32– 34, 40^. Furthermore, nNOS in the SR translocates to the caveolae to protect the myocardium from Ca ^2+^ overload and oxidative stress ^7, 33, 41^. Intriguingly, both eNOS and nNOS affect intracellular Ca ^2+^ handling in the myocytes, and eNOS mediates spontaneous Ca ^2+^ sparks and enhanced Ca ^2+^ transients in cardiac myocytes in response to increased preload (mechanical stretch) ^42^. Conversely, nNOS (but not eNOS) mediates the afterload-induced spontaneous Ca ^2+^ sparks ^43^. Spatial redistribution of NOSs is associated with both the changes of their activity and the shifting of the primary targets that underlie the mechanisms of myocardial function under stress. In essence, the translocation of nNOS may be beneficial in maintaining its activity to exert cardiac protection.

## Multifaceted mechanisms mediating the effects of neuronal nitric oxide synthase

It is generally accepted that *S*-nitrosylation (or *S*-nitrosation) and soluble guanylate cyclase (sGC)/cyclic guanosine monophosphate (cGMP)/protein kinase G (PKG)-dependent phosphorylation are the predominant mechanisms that mediate the effects of NO in biological systems ( Figure 1). The former mechanism involves post-translational modification of a thiol group in proteins by NO (transferring NO to cysteine residues, -SNO), and the latter implicates PKG-dependent phosphorylation of serine residues of the target proteins. *S*-nitrosylation is explicitly initiated by NO, but dinitrogen trioxide (N _2_O _3_), the nitrosonium ion (NO ^+^), peroxynitrite (ONOO ^−^), and SNO proteins are also able to deliver NO to the cysteine residues of the target proteins ^44, 45^. Protein-protein transfer of NO (trans- *S*-nitrosylation) is now known to represent one of the most important mechanisms of NO ^46^ ( Figure 1). In this process, the SNO “donor” proteins are referred to as nitrosylases. Trans- *S*-nitrosylation possesses advantages for effective interactions between proteins ^47^. Furthermore, transnitrosylation is important when NO bioavailability is limited in an oxidative and/or nitrosative stress environment, such as during ischaemic reperfusion. *S*-nitrosylation can be terminated by the action of denitrosylases (for example, *S*-nitrosoglutathione reductase and thioredoxin), with NADH and NADPH serving as electron donors to regenerate glutathione and thioredoxin ^48, 49^.

Various types of proteins are targeted by NO, which in turn triggers an array of signalling cascades depending on the properties of the target proteins, e.g. inhibition of protein phosphatase 2A/protein phosphatase 1 by NO leads to protein kinase A (PKA) and Ca ^2+^-calmodulin-dependent kinase II-dependent phosphorylation of downstream effector proteins such as phospholamban (PLN) ^50^, whereas sGC activation by NO in the myocardium of hypertensive rats causes cGMP/PKG-dependent phosphorylation of cTnI and cMyBPC ^34^. Conversely, phosphodiesterase 5 (PDE5) activation by NO/sGC/cGMP/PKG limits cytosolic cGMP, a negative feedback mechanism of NO regulation of cGMP in cardiac myocytes ^51^. In addition, by targeting cardiac oxidases, such as xanthine oxidoreductase ^52^, NADPH oxidase ^53, 54^, and mitochondrial reactive oxygen species (ROS) production ^55^, nNOS-derived NO controls intracellular oxidative status and ROS-dependent downstream effects in the myocardium. Cysteine residues are the targets of ROS to cause *S*-glutathionylation in the proteins ^56, 57^; ** therefore, *S*-nitrosylation by NO may “block” critical cysteine residues from irreversible oxidation under the conditions, such as increased oxidative stress. Consequently, post-transcriptional modifications downstream of NO change the effector proteins, altering their localisation, binding partners, activity, and, ultimately, function.

nNOS has also been demonstrated to produce H _2_O _2_ in the endothelium of large arteries, such as the aorta, and H _2_O _2_ mediates endothelium-dependent vascular relaxation ^58, 59^. Conversely, impairment of endothelial nNOS-derived H _2_O _2_ has been shown to worsen endothelial dysfunction in both atherosclerosis ^60, 61^ and hypertension ^62^, indicating a protective role of nNOS-derived H _2_O _2_ in the vasculature. Similarly, both eNOS-derived NO and nNOS-derived H _2_O _2_ contribute to acetylcholine stimulation of vasodilatation ^59^ by regulating similar downstream protein kinases and phosphatases ^63– 65^. In contrast, uncoupling of eNOS and nNOS (secondary to the deficiency of L-arginine, tetrahydrobiopterin [BH4] oxidation, or *S*-glutathionylation ^52, 66– 68^) results in the production of superoxide (O _2_^−^) instead of NO; under such conditions, eNOS and nNOS become the sources of oxidative stress for pathological progression in the myocardium.

Taken together, the mechanisms mediating the effect of NO are complex. *S*-nitrosylation, transnitrosylation, and sGC/cGMP/PKG-dependent phosphorylation provide major post-transcriptional modifications of NO. By producing H _2_O _2_, O _2_^−^, and the NO derivatives, NOSs also function as the upstream regulators of redox-dependent signalling.

## Effector targets of neuronal nitric oxide synthase maintaining cardiac contraction and relaxation during disease progression in the heart

### (I) Proteins involved in nitric oxide regulation of cardiac electrophysiology and intracellular Ca ^2+^ homeostasis

nNOS exerts its cardiac protection through the regulation of ion channels, modulating abnormal Ca ^2+^ homeostasis, mitochondrial function, and signalling pathways during pathological progression ^7, 8^ ( Figure 1). To fulfil the effects on cardiac electrophysiology and intracellular Ca ^2+^ homeostasis, nNOS regulates key ion channels and Ca ^2+^-handling proteins that participate in the process of electrical activity and excitation-contraction coupling of cardiac myocytes. In particular, nNOS has consistently been shown to reduce Ca ^2+^ influx through the L-type Ca ^2+^ channel (LTCC) ^69^, and its effect is potentiated in cardiac myocytes of female mice following post-ischaemia/reperfusion and significantly reduces ischaemia/reperfusion injury ^41^. In support of this, nNOS increases the vulnerability of the LTCC for Ca ^2+^-dependent inactivation in hypertensive cardiac myocytes ^70^ where intracellular Ca ^2+^ transient is increased secondary to nNOS-dependent myofilament Ca ^2+^ desensitisation ^34^. The effect of nNOS on the LTCC can be mediated by both *S*-nitrosylation and cGMP/PKG-dependent phosphorylation ^41, 71, 72^. Modulation of the LTCC by nNOS may prevent excessive intracellular Ca ^2+^ loading in cardiac myocytes under pathological threat. *S*-nitrosylation of the ryanodine receptor (RyR) by nNOS has been implicated in reducing diastolic Ca ^2+^ leak ^73^, increasing RyR open probability, and increasing contraction in cardiac myocytes ^74^. Therefore, nNOS protects against arrhythmogenesis by modulating Ca ^2+^ transients ^75, 76^. On the other hand, greater nNOS activity at the plasma membrane (subsequent to dissociation from the PMCA-containing complex) induces greater Na ^+^ influx through voltage-gated sodium channels (Nav1.5) via *S*-nitrosylation and enhances the susceptibility of the myocardium for long QT and arrhythmias ^38^. Potassium channels are also potential targets of nNOS through *S*-nitrosylation and/or cGMP/PKG-dependent phosphorylation ^77– 79^, which may play important roles in the regulation of cardiac electrophysiology and mechanical function in both healthy and diseased hearts.

nNOS-derived NO, or the formation of ONOO ^−^, can induce *S*-nitrosylation of the SR calcium ATPase (SERCA) both under basal conditions and with stimulation ^76, 80^ (for example, myocardial infarction). Inhibition of nNOS reduces *S*-nitrosylation of SERCA at basal level, and this is associated with reduced Ca ^2+^ uptake in the SR and decreased relaxation ^80^. However, the functional significance of this regulation under disease conditions remains to be determined. Alternatively, SERCA activity can be increased by nNOS via PKA-dependent phosphorylation of PLN secondary to nNOS-dependent inhibition of protein phosphatase 2A activity in left ventricular (LV) myocytes from normal mice ^50^. A recent report has shown that beta-adrenergic stimulation induces *S*-nitrosylation of PLN and increases its pentamerisation and the activation of SERCA. Whether the source of NO for the *S*-nitrosylation is from nNOS is not revealed in the study. However, nNOS-dependent PLN pentamerisation following beta-adrenergic stimulation are shown not to affect basal and beta-adrenergic phosphorylation of PLN. This is important because the results confirm that nNOS, either through *S*-nitrosylation under β-adrenergic stimulation or through phosphorylation secondary to the inhibition of protein phosphatases, promotes SERCA activity and exerts positive lusitropy in the myocardium. In addition, these results emphasise that *S*-nitrosylation and phosphorylation work in concert to mediate the effects of nNOS on cardiac function.

On the other hand, phosphorylation of PLN (Ser ^16^) is increased by nNOS through a cGMP/PKG-dependent mechanism independent of scavengers of ONOO ^−^, O _2_^−^, or PKA in cardiac myocytes stimulated by angiotensin II (Ang II) where nNOS is upregulated ^53^. Furthermore, phosphorylation of PLN (Ser ^16^) is increased in Ang II-induced hypertensive rat ventricular myocytes, but this response is independent of nNOS or cGMP/PKG signalling and exerts little effect on nNOS facilitation of myocyte relaxation ^34^. These results suggest that the modes of post-transcriptional modification that underlie the specific effects of nNOS are highly dynamic, and this may optimise its regulation of the downstream target proteins under various stimuli, including pressure overload.

### (II) Myofilament proteins are targeted by nitric oxide through S-nitrosylation and phosphorylation

A recent study from our own group has shown that nNOS-derived NO increases cGMP/PKG-dependent phosphorylation of cardiac troponin I (cTnI-Ser ^23/24^) and cardiac myosin binding protein C (cMyBPC-Ser ^273^) and promotes myocyte relaxation in the hypertensive heart through cGMP/PKG-dependent myofilament Ca ^2+^ desensitisation ^34^, indicating the involvement of myofilament proteins in nNOS-dependent responses in hypertensive myocardium. In fact, isobaric tag for relative and absolute quantitation (iTRAQ)-based quantitative proteomic analysis shows that nNOS affects the phosphorylation of almost 20 myofilament proteins in LV myocytes from the healthy heart and a similar number of distinct proteins in the hypertensive heart ^38^. These results indicate that myofilament proteins are the potential targets of nNOS that mediate faster relaxation in cardiac myocytes to reduce the mechanical load of the myocardium in hypertension. This is consistent with previous findings that exogenous NO donors facilitate myocardial relaxation via sGC and cGMP/PKG-dependent phosphorylation of cTnI and myofilament Ca ^2+^ desensitisation ^81^. A recent report has demonstrated that NO mimetics ( *S*-nitrosocysteine) reduce myofilament Ca ^2+^ sensitivity and myocardial contractility by inducing the *S*-nitrosylation of a number of myofilament proteins including actin, myosin, cMyBPC, and troponin C (cTnC) ^82^. More directly, both TnC-Cys35 and TnC-Cys84 are *S*-nitrosylated by beta-adrenergic stimulation and TnC-Cys84 is shown to be responsible for reduced myocardial Ca ^2+^ sensitivity in normal hearts. In contrast, *S*-glutathionylation of myofilament proteins in the hypertrophic myocardium increases myofilament Ca ^2+^ sensitivity and impairs relaxation ^83, 84^. These results strongly indicate that phosphorylation and *S*-nitrosylation (as well as oxidation) of myofilament proteins are the fundamental mechanisms that mediate the effects of nNOS in normal and diseased hearts.

### (III) Mitochondrial activity and biogenesis are dynamically regulated by nitric oxide

nNOS is regarded as the potential isoform that is expressed in the mitochondria to actively regulate cardiac metabolism ^85^. NO inhibits cytochrome c oxidase (complex IV) activity by competing with O _2_ and inhibits electron transfer of complex III (between cytochrome b and c) or NADH-dehydrogenase function at the level of complex I and increases mitochondrial production of O _2_^−^. Consequently, NO inhibits the mitochondrial respiration chain and reduces mitochondrial oxygen consumption ^86– 91^. As such, NO has generally been acknowledged as the negative regulator of mitochondrial activity and energy metabolism. This is seemingly counterintuitive to the cardiac protection of nNOS in the diseased heart or in the heart under stress because of the consensus that nNOS exerts protective roles. Nevertheless, conditional overexpression of nNOS in the myocardium has been associated with increased nNOS in the mitochondria and attenuation of mitochondrial ROS production and a reduction in oxidative stress following myocardial infarction ^55^. Although it remains to be confirmed, the modulation of oxidative stress by endogenous nNOS in diseased hearts can be a potential protective mechanism.

Emerging evidence shows that nNOS-derived NO plays essential roles in mitochondrial biogenesis ^92, 93^ to maintain or increase mitochondrial integrity and activity. For example, nNOS has been shown to be redistributed to the nucleus via α-syntrophin through its PDZ domain in a variety of cells, including myocytes ^37, 94^. Increased *S*-nitrosylation of nuclear proteins, including cAMP response element-binding protein (CREB), in turn, interacts with the promoter of the gene encoding peroxisome proliferator-activated receptor γ co-activator (PGC)-1α promoter, an essential component for mitochondrial biogenesis and nuclear respiratory factor 1 and mitochondrial transcription factor A ^37^. *S*-nitrosylation of nuclear proteins has also been ascribed to the trans- *S*-nitrosylation activity of glyceraldehyde 3-phosphate dehydrogenase (GAPDH) ^95^.

Additionally, NO has been implicated in cardiac energetics by affecting carbohydrate metabolism both within and outside of mitochondria. For example, NO stimulates glucose transport by activating upstream signalling pathways that result in increased amounts of the glucose transporter GLUT4 at the cell surface ^96^. Accordingly, inhibition of NOSs reduces the uptake of glucose and ATP production in skeletal muscle, both under basal conditions and during physical activity ^96– 99^. Moreover, NO has been implicated in the inhibition of the glycolytic enzyme GAPDH by means of *S*-nitrosylation ^100, 101^.

It should be noted that both nNOS and eNOS are required for mitochondrial activity, including biogenesis in many types of cells, such as myocytes ^92, 102, 103^. A typical example is that bradykinin inhibits mitochondrial oxygen consumption via eNOS in myocardial tissue ^104^ and that nNOS-derived NO remains unaffected. However, in the absence of nNOS, reduced NO bioavailability secondary to increased xanthine oxidase-derived O _2_^−^ limits the effect of eNOS in controlling mitochondrial oxygen consumption ^105^, indicating the interplay between eNOS and nNOS in the regulation of mitochondrial activity and cardiac metabolism. Detailed mechanisms of the interaction between eNOS and nNOS in mitochondria and its functional relevance in healthy and diseased hearts, however, remain to be determined.

## Nitric oxide, nitric oxide synthase, and cardiovascular therapy

Because of the importance of NO and its significance in the cardiovascular system, approaches that manipulate the bioavailability of NO in the myocardium are essential therapeutic strategies for the better treatment of cardiovascular diseases (CVDs). In fact, nitroglycerin, an organic nitrate that releases NO, has been used clinically in the treatment of CVD for more than 150 years (despite the fact that the effective molecule for the response, NO, was identified in the late 1970s ^1^). Enhanced acknowledgement of the mechanistic insights into NO signalling, exogenous versus endogenous NO sources, the maintenance and the degradation of NO, and the properties of NOSs as well as modern technology enables novel approaches to increase NO bioavailability in target tissues for the desired responses. In principle, enhancement of NO and its signalling can be achieved through three routes: increase exogenous and endogenous sources to promote NO production, reduce NO metabolism/degradation, and stimulate downstream signalling of NO.

A number of strategies are used to promote NO formation. For example, inhaled NO is registered to be applied to newborn babies with persistent pulmonary artery hypertension ^106, 107^ to support ventilation-perfusion match and to prevent systemic ischaemia. Nitrite can be similarly applied; in fact, the effectiveness of oral, inhaled, and intravenous nitrite on a number of CVDs (such as pulmonary artery hypertension [PAH], peripheral vascular diseases, myocardial infarction, and cerebral vasospasm after subarachnoid haemorrhage) are under study with promising prospects on some occasions ^108– 110^. Delivering organic and inorganic nitrate and nitrite to amplify systematic or local NO through nitrate–nitrite–NO and the nitrate–nitrite–NO–fatty acid pathways are probably the most active area under investigation experimentally and in the clinic ^10^. So far, a number of putative precursors of NO (nitroxyl [HNO], S-nitrosothiols, sodium nitrite, sodium nitrate, nitrated fatty acids, and nitrate from beetroot juice and green leaves, such as spinach, etc.) have been identified and are under development. Dietary consumption of NO precursors is a cheap, safe, and effective way of nitrate delivery; programming of a suitable diet regime for vulnerable populations will be important to reduce the cardiovascular risks as well as the economic burden on national healthcare systems. The relationship between the daily consumption of nitrate and cardiovascular events is noticeable. For example, high fruit and green vegetable intake in a Japanese population historically known to have low rates of CVD (daily consumption of nitrate >1,100 mg/60 kg) is associated with greater circulating nitrate and nitrite ^111^ compared to those in the US and Europe, where average daily nitrate consumption ranges from 40–100 mg and 30–180 mg, respectively, and the rates of CVD are high ^112, 113^. Moreover, the consumption of “healthy” fats, as in the Mediterranean diet, in the form of unsaturated fatty acids such as oleic and linoleic acid (to form nitrated fatty acids), is beneficial in preventing the development of CVD and reduces the risk factors ^114^. Notably, nitrite reduction to NO preferentially occurs in the presence of hypoxia and acidosis, during physical exercise, at the time when cardiac muscle needs NO the most.

Alternatively, supplementation of NO substrates, e.g. arginine, L-citrulline, and BH4 (a co-factor of NOS), and inhibition of arginase and asymmetric dimethylarginine (an endogenous NOS inhibitor) are the necessary strategies to increase NO through promoting NOS activity ^11^. Statins and nebivolol or carvedilol (new-generation beta1-adrenergic receptor blockers) exert anti-adrenergic responses via the stimulation of the beta3-adrenergic receptor and increasing NOS production of NO ^115– 118^. Targeting NOS is advantageous in mediating the specific downstream signalling of NOSs in the compartments.

Attempts to prevent NO reduction and inhibition of NOS uncoupling are also important in maintaining or increasing cytosolic NO. Decreasing the formation of ROS using blockers of angiotensin-converting enzyme (ACE), angiotensin I type 1 receptor (AT1R), or NADPH oxidases (NOXs) or reducing ROS by using antioxidants and scavengers are the putative mechanisms to reduce NO “sink” and therefore maintain or increase NO level ^11^. However, the complex NOX isoforms and the redox–nitrosol network weaken the effectiveness of the developed drugs in clinical use. Indeed, our recent results indicate that NOX and ROS are upstream regulators of cardiac nNOS protein and activity downstream of Ang II and AT1R ^119^. Furthermore, Ang II type 2 receptor (AT2R) mediates Ang II enhancement of nNOS protein expression via ROS activation of eNOS activity ^119^. As such, it is wrong to simply assume that NO level can be increased by using ACE and AT1R inhibitors. The development of selective NOX inhibitors and specific ROS-manipulating drugs that do not affect NOS protein should be pursued, and the effect of NOX and ROS on nNOS protein expression in the myocardium should be taken into consideration.

Stimulation of the downstream signalling pathway of NO is an improved strategy to target the effector proteins directly, bypassing the complex scenario of NOSs and the redox–nitrosol network. Synthetic benzyl indazole compound YC-1, the oral sGC stimulators riociguat and vericiguat, and atrial, brain, and C-type natriuretic peptides are in use to increase cellular cGMP ^11^. Inhibition of a negative regulator of cGMP, PDE5 (e.g. ** using sildenafil), is another therapeutic approach to stimulate cGMP/PKG signaling ^120– 122^. Stimulation of the PKG-dependent pathway has been shown to exert potent protective effects in a wide array of cardiovascular disease models, including hypertension, PAH, heart failure, haemolytic anaemia, and infarct-reperfusion injury ^120– 124^. However, the application of the drugs in a large cohort of patients with CVD show minor responses to the treatment, suggesting further investigation on cGMP enhancers is still needed.

Primary *S*-nitrosothiols (RSNOs) are the endogenous NO carriers and donors and have emerged as platforms for the localised delivery of NO, which mediates *S*-nitrosation-dependent mechanisms ^125^. *S*-nitrosoglutathione (GSNO) is one of the main RSNOs and is a central intermediate in the formation and degradation of cellular *S*-nitrosothiols with potential therapeutic applications ^125^. So far, GSNO has not been implied in pharmaceutical composition owing to the fast decomposition in aqueous solutions. To sustain the bio-available NO donors and optimise their kinetics and to control the delivery of NO to targeted tissues or proteins, various biomaterial-based carriers (microparticles and nanoparticles) are under development ^126^.

Taken together, a number of validated ways have been developed to increase systemic and local NO levels and are promising in mediating the beneficial effects in CVD. However, in order to translate the research innovations into the application to a large population, more research is necessary, with special attention to the specificity and effectiveness, i.e. nitrate/nitrite regime in the diet and strategies of increasing nNOS (as well as eNOS) and improving NO-effector interactions in CVD settings. NO-dependent therapy holds promise as a therapeutic platform for CVD in humans.

## Future perspectives

Our understandings of the NO and NOSs that regulate myocardial contraction, relaxation, and pathological signalling are advanced, but the dynamic paradigm in the myocardium under stress is not clearly presented. NO from both exogenous and endogenous sources supply the bioavailable NO in the myocardium, and the level and effectiveness of NO is determined by multiple regulation mechanisms including daily consumption of NO precursors, nitrate from skeletal muscles, NO production through the entero-salivary NO pathway (oral and gut microbiome function as essential regulators) and from NOSs as well as redox environment and the nature and the abundance of target proteins. In general, NO regulates downstream effector proteins through three mechanisms (sGC/cGMP/PKG-dependent phosphorylation, *S-*nitrosylation, and transnitrosylation) and the numbers and types of effectors regulated by NO are diverse. As such, modification of these effectors by NO subsequently triggers an array of signalling cascades that lead to different physiological and pathological consequences. By and large, NO and its downstream signalling pathway exert potent cardiovascular protection; however, translational research of NO and NOS that are applicable for CVD and therapeutic efficiency using an NO-dependent regime are still far from satisfactory ^11^. Further research needs to be carried out aiming to increase the specificity of NO-effector interaction at the site of relevant proteins. Better design of a dietary program and the promotion and targeting of nNOS and eNOS in specific locations with the effector target(s) of importance will maximise the effectiveness of NO application in cardiovascular physiology and pathology.
